# Abundant protein phosphorylation potentially regulates Arabidopsis anther development

**DOI:** 10.1093/jxb/erw293

**Published:** 2016-08-16

**Authors:** Juanying Ye, Zaibao Zhang, Chenjiang You, Xumin Zhang, Jianan Lu, Hong Ma

**Affiliations:** State Key Laboratory of Genetic Engineering and Collaborative Innovation Center of Genetics and Development, Institute of Plant Biology, School of Life Sciences, Fudan University, Shanghai 200433, China

**Keywords:** Anther development, Arabidopsis, mass spectrometry, phosphoproteomics

## Abstract

As the male reproductive organ of flowering plants, the stamen consists of the anther and filament. Previous studies on stamen development mainly focused on single gene functions by genetic methods or gene expression changes using comparative transcriptomic approaches, especially in model plants such as *Arabidopsis thaliana*. However, studies on Arabidopsis anther protein expression and post-translational modifications are still lacking. Here we report proteomic and phosphoproteomic studies on developing Arabidopsis anthers at stages 4–7 and 8–12. We identified 3908 high-confidence phosphorylation sites corresponding to 1637 phosphoproteins. Among the 1637 phosphoproteins, 493 were newly identified, with 952 phosphorylation sites. Phosphopeptide enrichment prior to LC-MS analysis facilitated the identification of low-abundance proteins and regulatory proteins, thereby increasing the coverage of proteomic analysis, and facilitated the analysis of more regulatory proteins. Thirty-nine serine and six threonine phosphorylation motifs were uncovered from the anther phosphoproteome and further analysis supports that phosphorylation of casein kinase II, mitogen-activated protein kinases, and 14-3-3 proteins is a key regulatory mechanism in anther development. Phosphorylated residues were preferentially located in variable protein regions among family members, but they were they were conserved across angiosperms in general. Moreover, phosphorylation might reduce activity of reactive oxygen species scavenging enzymes and hamper brassinosteroid signaling in early anther development. Most of the novel phosphoproteins showed tissue-specific expression in the anther according to previous microarray data. This study provides a community resource with information on the abundance and phosphorylation status of thousands of proteins in developing anthers, contributing to understanding post-translational regulatory mechanisms during anther development.

## Highlight

Identification of over 1600 phosphoproteins and analysis of phosphorylation site motifs provide strong support for extensive protein phosphorylation and the potential regulatory roles for such modifications during anther development.

## Introduction

In flowering plants, the development of the male reproductive organ (stamen) is a complex process involving anther primordium initiation and early cell divisions, differentiation of anther tissues, generation of haploid microspores, as well as formation and elongation of filaments. A mature anther typically has four lobes; each lobe contains the central microspore mother cells (Ms) and somatic tissues, which include four separate sporophytic cell layers, the epidermis, endothecium, middle layer, and tapetum. Anther development in Arabidopsis can be divided into two phases and 14 stages based on the morphological changes of the tissue and cells visualized by light microscopy (Sanders *et al.*, 1999; Ma, 2005). During phase I, including stages 1–7, archesporial cells undergo differentiation and division to establish the mature anther morphology, and microspore mother cells form haploid spores through meiosis. During phase II, consisting of stages 8–14, the anthers are enlarged and pushed upward by filament extension, while sperm cells are formed and pollen grains mature. Then anther tissues degenerate and anther dehiscence releases pollen grains for delivery onto the stigma.

Recent genetic and biochemical evidence reveals that numerous regulatory factors, such as protein kinases (especially receptor-like kinases) and transcription factors (TFs), play crucial roles in anther development during different stages (Chang *et al.*, 2011; Zhang and Yang, 2014). *EXCESS MICROSPOROCYTES1* (*EMS1*, also known as *EXTRA SPOROGENOUS CELLS* [*EXS*]), encoding a receptor-like kinase, was the first such gene shown to play critical roles in determination of anther cell fate during early stages of anther development (Canales *et al.*, 2002; Zhao *et al.*, 2002). Subsequently, multiple lines of evidence supported the idea that TAPETAL DETERMINANT1 (TPD1) acts as the extracellular ligand of EMS1 during early anther development (Yang *et al.*, 2003, 2005; Jia *et al.*, 2008). *SOMATIC EMBRYOGENESIS RECEPTOR LIKE KINASE1 (SERK1*) and *SERK2*, two functionally redundant genes encoding receptor-like kinases, were found to be important for tapetum formation (Albrecht *et al.*, 2005; Colcombet *et al.*, 2005) and these two proteins might form heterodimers with EMS1 and perform functions in a similar manner as BRASSINOSTEROID INSENSITIVE1 (BRI1) and BRI1activation kinase (BAK1) in the brassinosteroid (BR) signaling pathway. In addition, *BARELY ANY MERISTEM1* (*BAM1*) and *BAM2*, encoding receptor-like kinases similar to CLAVATA1, are required for the specification of somatic cells during early stages of anther development (Hord *et al.*, 2006). Another receptor kinase, *RECEPTOR-LIKE KINASE2* (*RPK2*) is needed for the cell fate specification of the middle layer during the differentiation of secondary parietal cells into endothecium and middle layer cells (Mizuno *et al.*, 2007). *ERECTA* (*ER*), *ERECTA-LIKE1* (*ERL1*) and *ERL2* encode leucine-rich-repeat receptor-like kinases and mitogen-activated protein kinase (MAPK) 3 and MAPK6 function in similar fashion to regulate normal anther lobe formation and anther cell differentiation (Hord *et al.*, 2008). Two mutants in the gene for the brassinosteroid receptor BRI1, *cpd* and *bri1-116*, were demonstrated to be defective in both early anther development (tapetum development) and later exine pattern formation and pollen release (Ye *et al.*, 2010*b*). Also, phosphoserine phosphatase PSP1 is essential for male gametogenesis through affecting pollen and tapetum development in Arabidopsis (Cascales-Minana *et al.*, 2013; Flores-Tornero *et al.*, 2015). The functional importance of these proteins predicted to have protein kinase activities strongly suggests that protein phosphorylation might be crucial for normal anther development, although direct evidence for protein phosphorylation is generally elusive.

Transcriptional regulation is the most common and fundamental regulation during development. In addition, the activities of transcriptional factors (TFs) are often affected by post-translational modification, which coordinates various cellular processes. Reversible protein phosphorylation through protein kinases and phosphatases is one of the most widespread and important post-translational modifications in eukaryotes, often affecting the transcriptional regulatory network through phosphorylating or dephosphorylating TFs. For instance, in Arabidopsis phosphorylation of a WRKY TF (WRKY33) by MAPK3/MAPK6 is required for pollen development and function (Guan *et al.*, 2014). Also, the Arabidopsis AKIN10 protein delays flowering by inactivating a TF, IDD8, through protein phosphorylation (Jeong *et al.*, 2015). Phosphorylation of a basic leucine zipper (bZIP) TF, FD, by calcium-dependent protein kinases is crucial for its interaction with the florigen FT (Kawamoto *et al.*, 2015). Furthermore, phosphorylation of the Arabidopsis TF BES1/BZR2 by BIN2 might hamper the nucleus transport of BES1/BZR2, thus reducing the expression of downstream key genes involved in anther and pollen development (Ye *et al.*, 2010*b*; Wang *et al.*, 2011).

Many other genes have been implicated in anther development through transcriptomic analysis using microarray or next generation RNA sequencing techniques (Feng *et al.*, 2012; Ma *et al.*, 2012). The expression levels of messenger RNA cannot reveal the abundance of corresponding protein products; moreover, post-translational modification (PTM) of proteins also requires specific methods to be detected. PTMs of proteins, especially reversible protein phosphorylation, triggered by protein kinases and phosphatases, are important for regulating protein functions, such as signal transduction (Mithoe and Menke, 2011).

Proteomics analysis using mass spectrometry has emerged as a powerful technology for characterizing proteins and protein PTMs, including those from pollen. Recently the Grossniklaus group performed a proteomic profile of mature pollen and identified 3200 proteins approximately one-third of which were not detected from transcriptomic analysis (Grobei *et al.*, 2009). In addition Mayank *et al.* (2012) identified 598 phosphoproteins from the mature pollen grains. Another phosphoproteomic study identified 139 phosphoproteins from mature pollen grains and germinating pollen in tobacco (Fila *et al.*, 2012). Also, Wang *et al.* (2014) performed a large scale phosphoproteomic analysis using rice pistil tissue and identified 2347 phosphorylation sites from 1588 phosphoproteins.

However, similar proteomic analysis has not been conducted for the Arabidopsis anther, and thus the protein expression and phosphorylation profiles during Arabidopsis anther development remain unknown. A systematic proteomic/phosphoproteomic study of the Arabidopsis anther would complement the extensive molecular genetic studies of genes encoding protein kinases, and provide valuable information for further understanding the molecular mechanisms controlling anther development at protein expression and post-translational regulation levels. Therefore, we performed a large-scale unbiased, global analysis to map the phosphorylation events that occur in the developing anther. Here, we took advantage of the high accuracy of mass spectrometry to explore the global protein expression level and protein phosphorylation status of two phases of developing anthers, one near the time of meiosis (stages 4–7) and another close to maturity (stages 8–12). The proteomic and phosphoproteomic results reported here provide a global survey on protein expression and phosphorylation profile during Arabidopsis anther development. The results can facilitate the understanding of physiological functions underlying Ser/Thr/Tyr phosphorylation and signaling networks in anthers and related biological process.

## Materials and methods

### Plant materials and anther collection

*Arabidopsis thaliana* [ecotype Columbia (Col)] was grown in Metro-Mix 200 soil under long-day conditions (16h light–8h dark) in a growth chamber at a constant 22 °C. We selected 3- to 4-week-old plants to collect anthers at stages 4–7 and 8–12 as previously described (Wijeratne *et al.*, 2007). Anthers were isolated on a glass slide under a stereoscopic microscope, immediately frozen in liquid nitrogen and stored at −80 °C until use. For stage 8–12 samples, approximately 2000 anthers were collected, while approximately 8000 anthers were collected for stage 4–7 samples.

### Total protein extraction and trypsin digestion

Arabidopsis anthers were ground to fine powder in liquid nitrogen, and suspended with buffer containing 6M urea, 2M thiourea and 100mM NH_4_HCO_3_ (final concentration). The suspension was sonicated for 15min (2s sonication at 5s intervals) and the supernatant was collected by centrifugation at 20 000×*g* for 20min. The proteins reduction, alkylation and digestion were performed as described previously (Ye *et al.*, 2010*a*).

### Phosphopeptide enrichment by TiO_2_ affinity chromatography

The peptide mixture was acidified to remove the pigments and other impurities. The supernatant was collected and lyophilized. Phosphopeptide enrichment was performed as described before (Thingholm *et al.*, 2006).

### Nano ultra performance liquid chromatography–tandem mass spectrometry analysis

Liquid chromatography–tandem mass spectrometry (LC-MS/MS) analysis was performed using an LTQ-Orbitrap Elite (Thermo Fisher Scientific) coupled with an Easy nano-LC 1000. The LC solvents were 0.1% formic acid in H_2_O (solvent A) and 0.1% formic acid in 90% acetonitrile (solvent B). The peptide separation was accomplished by three-step elution: 2–35% solvent B for 200min, 35–90% solvent B for 10min, 90% solvent B for 5min, 90−2% solvent B for 2min, and 2% for 13min. Up to 15 of the most intense peptide ions (>5000 counts) were selected and fragmented by MS/MS using multistage activation in the linear ion trap.

### Database search and data analysis

Raw data were processed by Proteome Discoverer software (Version 1.4, Thermo Fisher Scientific, Germany) and searched against the Arabidopsis protein sequence database (3 August 2010; 33 410 sequences) using an in-house Mascot server (Version 2.3.02, Matrix Science, London, UK). The following parameters were specified in the protein database searches: only tryptic peptides with up to two missed cleavage sites were allowed as done previously (Meissner *et al.*, 2013; Deeb *et al.*, 2014); 10 p.p.m. mass tolerances for MS and 0.6Da for MS/MS fragment ions; carbamidomethylcysteine as a fixed modification; and protein N-acetylation, oxidized methionine, and phospho_STY (serine, threonine, and tyrosine) were permitted as variable modifications (Ye *et al.*, 2010*a*). Peptide identification was achieved by using the following requirements: peptide confidence was set as high (peculator value <0.01). Identified peptides were further validated with Target Decoy PSM validator and phosphorylation sites were evaluated with phosphoRS3.0. The protein abundance was estimated according to the exponentially modified protein abundance index (emPAI) value provided by the Mascot search engine. EmPAI is a measurement that converts the total sample amount to the absolute amount of each protein in the sample according to the ion current of all observable peptides of the protein.

Functional classification of identified proteins was facilitated by online Mapman analysis (Usadel *et al.*, 2006). A list of kinases for potential substrates was generated through PhosAtDB (http://phosphat.uni-hohenheim.de/) (Zulawski *et al.*, 2013). Predicted kinase–substrate networks were illustrated by Cytoscape 2.8 software.

## Results

### Proteins and phosphoproteins identified in anthers

Developing anthers can be divided into two phases according to the morphological features: stages 1–7 (pre-meiosis and meiosis) and 8–14 (post-meiosis, corresponding to microspore development and pollen maturation of the gametophyte). As stages 1–3 anthers are too small to dissect from the floral buds, we collected anthers at stages 4–7 (phase I) for the first sample group. In addition, we sampled anthers at stages 8–12 (anthers before dehiscence; phase II), as the late phase of anther development.

**Fig. 1.**
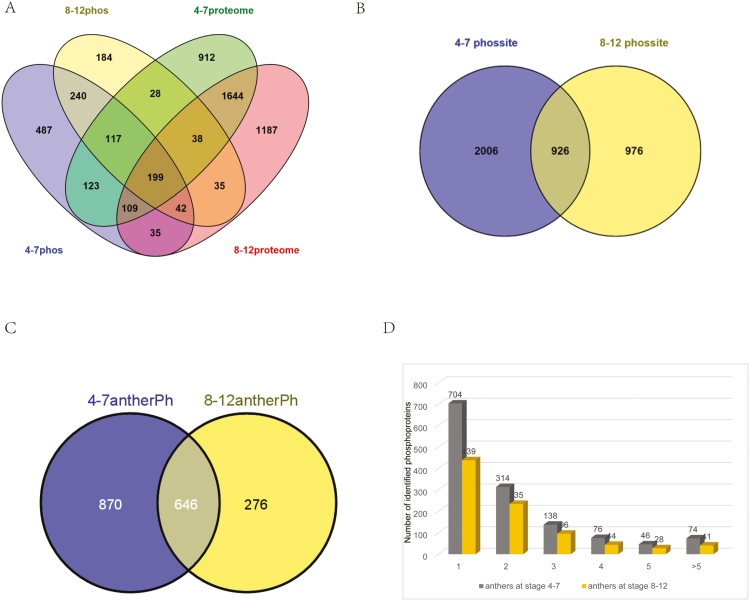
An overview of the proteins and phosphoproteins identified in the proteome and phosphoproteome of developing anthers. (A) Overlap of the identified proteins from proteomic and phosphoproteomic analyses of two phases. (B) Overlap of the phosphorylation sites identified from two phases. (C) Overlap of the phosphoproteins identified from two phases. (D) Distribution of the identified phosphorylation sites per protein.

Proteomic and phosphoproteomic analyses were performed in parallel for the two samples. One-tenth of the protein extracts was used to determine the protein expression patterns, while the remaining nine-tenths was used to characterize the protein phosphorylation profiles. In total, we identified 20 815 tryptic peptides (including 3954 phosphopeptides; Supplementary Table S1 at *JXB* online), which corresponded to 5380 proteins (including 1637 phosphoproteins; Fig. 1A). Mapman analysis of the identified total proteins revealed that 14 functional categories were remarkably enriched (*P*<1.0×10^–10^) compared with the whole Arabidopsis proteome (Supplementary Fig. S1).

### Phosphorylation site assignment of identified phosphoproteins in anther phases I and II

In this study, we obtained 3954 non-redundant phosphopeptides (2776 from phase I and 1714 from phase II; Supplementary Table S1) containing 3908 phosphorylation sites (2932 from phase I and 1902 from phase II; Fig. 1B), which were assigned to 1637 phosphoproteins (1352 from phase I and 883 from phase II; Supplementary Table S2). Because the phosphopeptides were enriched prior to MS analysis, it was not possible to estimate the fraction of a protein being phosphorylated. A comparison of the phosphorylation sites and their corresponding phosphoproteins identified in the anthers from two phases showed that 646 phosphoproteins and 926 phosphorylation sites were identified in both samples; 870 and 276 phosphoproteins as well as 2006 and 976 phosphorylation sites were uniquely identified in the samples from phases I and II, respectively (Fig. 1B, C).

The number of phosphorylation sites in a specific phosphoprotein varied considerably in both samples. Although most proteins had only a few detected phosphorylated sites, a number of proteins were identified with more than 15 phosphorylation sites (Supplementary Table S3). An extraordinarily large number (46) of phosphorylation sites were identified for AT5G07530.1, an oleosin domain-containing glycine-rich protein, in phase II. It is expressed specifically during flower stages 10–12 and deposited on the pollen wall (Mayfield and Preuss, 2000). The number of phosphoproteins with different numbers of phosphorylation sites in two samples are shown in Fig. 1D, with around 50% possessing only one phosphorylation site, and more than 20% in both samples having two sites. Approximately another 8% of the identified phosphoproteins in both samples contained five or more phosphorylation sites, including 27 TFs (24 in phase I and 14 in phase II; Supplementary Table S3), suggesting extensive regulation of the transcriptional networks by protein phosphorylation.

Among the 2932 identified phosphorylation sites in phase I and 1902 sites in phase II, 89.26% (2617/2932) and 89.59% (1704/1902) were on serine (pSer), 9.55% (280/2932) and 10.41% (198/1902) on threonine (pThr), and 0.89% (35/2932) and 0% on tyrosine (pTyr), respectively (Supplementary Fig. S2). These findings are consistent with previous results in mammals and plants (Olsen *et al.*, 2006; Nakagami *et al.*, 2010).

### Phosphopeptide enrichment prior to LC-MS analysis promoted the identification of low-abundance proteins and regulatory proteins

**Fig. 2.**
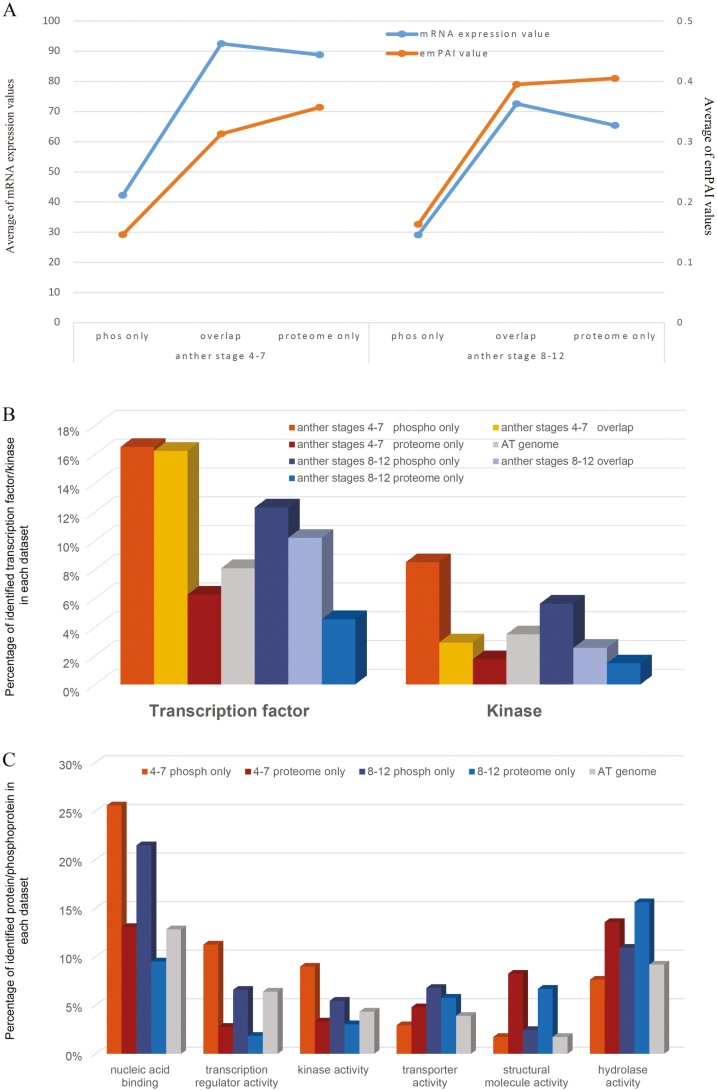
Enrichment analysis of low-abundance and regulatory proteins in the proteome and phosphoproteome of anthers in phases I and II. (A) Average abundance of proteins identified in three datasets (proteins identified only in the phosphoproteomic analysis, proteins identified in both analyses and proteins identified only in the proteomic analysis) in phases I and II. (B) Distribution of TFs and kinases/phosphatases in three datasets, the phosphoproteome only, both the proteome and phosphoproteome and the proteome only. (C) Over/under-represented gene ontology (GO) molecular function categories in the proteome and phosphoproteome of two phases. Regulatory proteins are dominantly phosphorylated, especially in anther phase I.

By comparing the results from proteomic and phosphoproteomic analyses, the identified proteins were divided into three subsets: only in phosphoproteomic analysis, only in proteomic analysis and in both analyses (Supplementary Fig. S3). After phosphopeptide enrichment, 806 and 569 additional proteins were identified in anther phases I and II, respectively. According to the exponentially modified protein abundance index (emPAI) value, which is commonly used to estimate protein abundance from large-scale identification data by LC-MS/MS, provided by the Mascot search engine (Ishihama *et al.*, 2005), the abundance of proteins identified only in phosphoproteomic analysis was more than two times lower than that in either only proteomic analysis or both analyses (Fig. 2A). This suggests that phosphopeptide enrichment prior to LC-MS analysis promotes the identification of low-abundance proteins and provides additional information for proteomic analysis, thus increasing the identification coverage of proteomic analysis.

Other than structural proteins, most of the functional proteins, especially regulatory proteins (mainly including TFs, protein kinases/phosphatases) are not abundant *in vivo*, although they play crucial roles in development and other processes. Therefore, we calculated the percentages of the regulatory proteins in the three datasets for two samples. The percentages of TFs and kinases in phosphoproteomic analyses were about two times higher than that in proteomic analyses (Fig. 2B). Further agriGO analyses (Du *et al.*, 2010) on molecular functions of the identified proteins in three datasets revealed that proteins annotated to have transcriptional regulator activity and binding activity (mainly DNA binding; TFs) were greatly over-represented in phosphoproteomic datasets, while proteins involved in structural molecule activity, catalytic activity and binding activity (mainly nucleotide binding) were significantly enriched in proteomic datasets (Fig. 2C). Therefore, our phosphoproteomic analysis enabled the identification of low-abundance proteins, which are very difficult to identify using conventional proteomic analysis, thereby facilitating the analysis of more regulatory proteins than otherwise would be possible.

### Mapman analysis of proteins and phosphoproteins in anther phases I and II

**Fig. 3.**
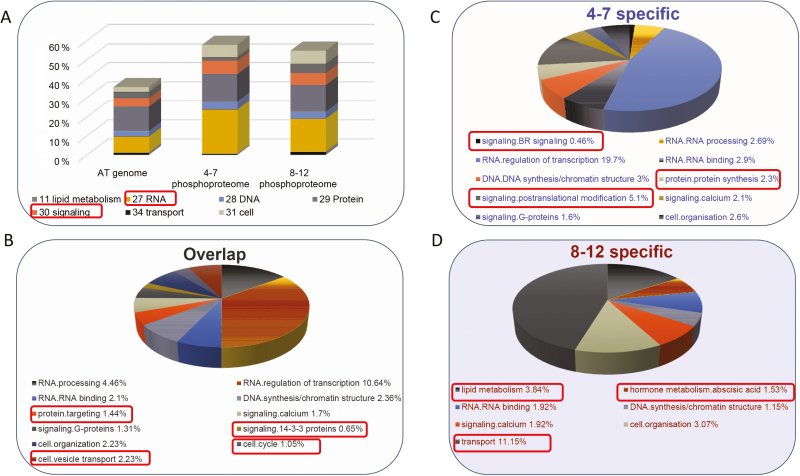
Characteristics of proteins identified in the phosphoproteome of anther phases I and II. (A) Mapman analysis of the phosphoproteins identified in anther phases I and II. Only the significantly over/under-represented bins (primary Mapman bins with *P<*1.0×10^–2^) are included. (B) Mapman analysis of the phosphoproteins only identified in phase I. (C) Mapman analysis of the phosphoproteins identified in both phases. (D) Mapman analysis of the phosphoproteins only identified in phase II. Only the bins (secondary Mapman bins) with *P<*1.0×10^–2^ are included.

To obtain a global overview of functional classification of the anther proteins and phosphoproteins identified from the two anther phases, we performed Mapman analysis, a functional categorization enrichment method for plant omics data analysis (Usadel *et al.*, 2006), for the proteomic and phosphoproteomic datasets of both anther phases, and the Arabidopsis proteome was used as background. The results showed that most of the energy metabolic pathways were over-represented in the proteomic datasets for both anther phases (Supplementary Fig. S4). On the other hand, the regulatory pathways were greatly under-represented in both anther phases, especially in anther phase II (Supplementary Fig. S4). However, Mapman analyses showed opposite results for the phosphoproteomic datasets; the regulatory pathways were highly over-represented in both anther phases (Fig. 3A). The results further highlighted that many of the phosphoproteins identified in both anther phases are likely involved in regulatory pathways.

Anther development is a dynamic process, and thus the regulatory mechanisms involved in different anther stages may vary. A comparative analysis was performed on the identified phosphoproteins from two anther phases to assess whether the proteins regulated by phosphorylation are distinct between the two anther phases. As mentioned above (Fig. 1C), 646 phosphoproteins were identified in both anther phases, 870 phosphoproteins were only identified in phase I and 276 only in phase II.

Further functional enrichment was performed by Mapman analysis on three datasets: phase I specific, phase II specific and shared by the two phases. The results revealed that four functional categories were over-represented in all three datasets, including ‘RNA/RNA binding’, ‘DNA/DNA synthesis/chromatin structure’, ‘signaling/calcium’ and ‘cell organization’. Three functional categories were over-represented in both phase I-specific and the shared phosphoproteomic datasets, including ‘RNA/RNA processing’, ‘RNA/regulation of transcription’ and ‘signaling/G-proteins’ (Fig. 3C). Furthermore, three categories were solely enriched in phase I-specific datasets, including ‘signaling/BR signaling’, ‘signaling/posttranslational modification’ and ‘protein/protein synthesis’ (Fig. 3B). The latter results suggest that phosphorylation on proteins involved in BR signaling and post-transcriptional modification might regulate the activities of these proteins during early anther development. Four categories, including ‘protein targeting’, ‘cell/vesicle transport’, ‘signaling/14-3-3 proteins’ and ‘cell cycle’, were enriched in the shared dataset, indicating that phosphorylation of proteins involved in these categories are important for both phases. In addition, ‘lipid metabolism’, ‘transport’ and ‘hormone metabolism/abscisic acid’ were greatly enriched in the phase II-specific dataset (Fig. 3D). During phase II, anthers mainly support pollen maturation and anther somatic layers degenerate, and thus the proteins involved in these processes need to be active. The biological functions of phosphoproteins enriched in Mapman analysis of the phase II-specific dataset were highly related to the processes that occur during phase II, and therefore we propose that phosphorylation may modulate the activities of the proteins involved in the processes of lipid metabolism, transport and hormone metabolism/abscisic acid during anther development phase II.

### Preferential phosphorylation of residues located in variable protein regions among family members, but conserved across eudicots and angiosperms

**Fig. 4.**
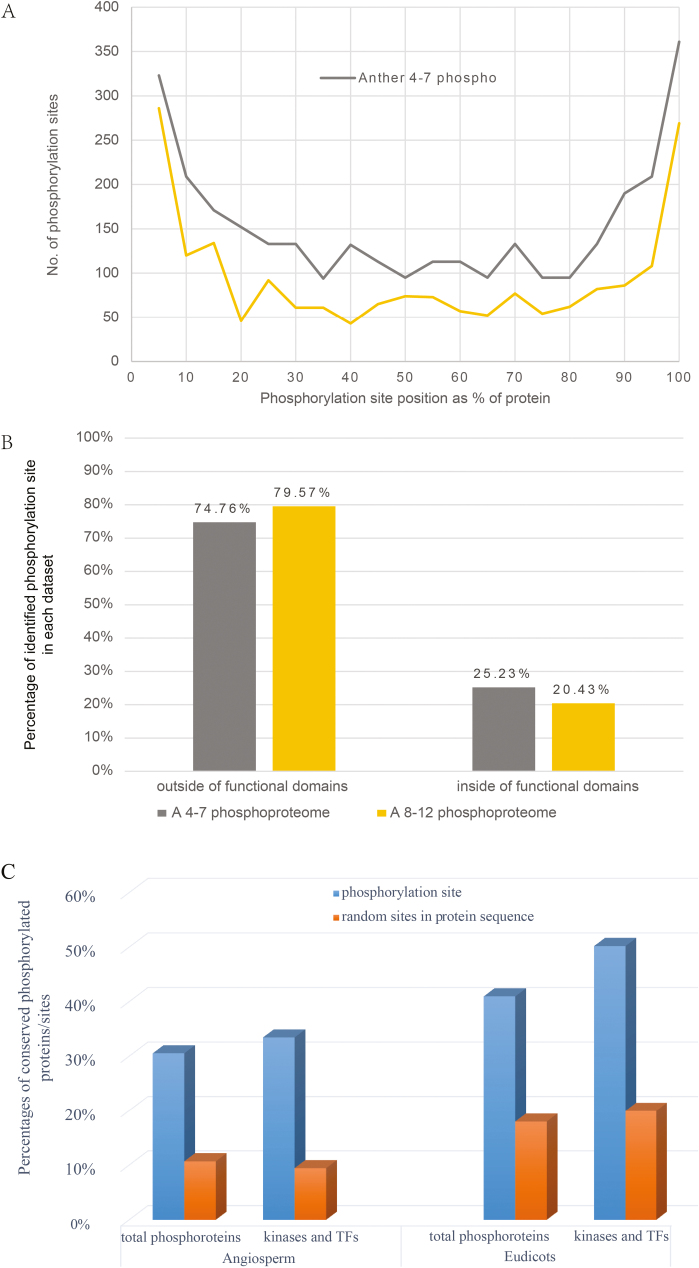
Analysis of identified phosphorylation sites. (A) Positional distribution of the identified phosphorylation sites in protein sequences. In this analysis, each protein sequence was evenly divided into 100 fractions and every five fractions were set as one unit, and then the number of phosphorylation sites within each unit was calculated. Phosphorylation preferentially occurred in the protein terminus (N-terminus or C-terminus). (B) Comparison of the phosphorylation sites located inside and outside the region of the functional domains (predicted by Pfam) of the phosphoproteins. Phosphorylation preferentially occurred in the unconserved linker regions. (C) Conservative analysis of phophorylation sites across eudicots and angiosperm.

Proteins usually contain functional domains, which define protein families; whereas regions outside these conserved domains, including N- and C-terminal regions and linker regions between domains, are often variable among family members. Nevertheless, such variable regions can still be conserved between orthologs from different organisms, such as angiosperms. To assess whether there is any preference of the phosphorylation sites in specific protein region, the distribution of 2932 and1902 identified phosphorylation sites from phase I and phase II, respectively, were analysed. In particular, a protein sequence was evenly divided into 100 fractions and every five fractions formed one unit, and then the number of phosphorylation sites in each unit was calculated. As shown in Fig. 4A, 22.74% (703/3092) and 28.39% (540/1902) of the phosphorylation sites were located in the N-terminus of protein (position from 0 to 15% of protein sequences) in phases I and II, respectively. In addition, 24.58% (760/3092) and 24.34% (463/1902) of the identified phosphorylation sites were located in the C-terminus of protein (position from 85 to 100% of protein sequences) in phases I and II, respectively. The results suggest that the N- and C-terminal regions are preferentially phosphorylated, possibly because the N- and C-termini are more likely to be exposed outside of the functional domains and are more flexible than the internal regions of the proteins, and thus more accessible to kinases.

To further assess whether the phosphorylation sites are located in functional domains or other regions, all identified phosphoproteins were subjected to online Pfam analysis (http://pfam.xfam.org/search#searchBatchBlock) to retrieve the domain information. A comparison of the positions of the identified phosphorylation sites with the positions of functional domains predicated by Pfam analysis showed that 74.76% and 79.57% of the phosphorylation sites were localized outside the functional domain in phases I and II, respectively, whereas only 25.23% and 20.4% of them were located inside functional domains (Fig. 4B). It is possible that the number of the phosphorylation sites within conserved Pfam domains was underestimated, since the same phosphopeptide matching different family members was counted as one.

Comparative phosphoproteomic analyses have demonstrated that protein phosphorylation is evolutionarily and functionally conserved across prokaryotes and eukaryotes (Boekhorst *et al.*, 2008; Macek *et al.*, 2008). However, from the above results most of the phosphorylated residues are localized in the variable terminal of linker regions outside known functional domains. To assess whether the phosphorylation sites identified in our experiments are conserved between organisms, we compared the phosphorylated residues and their non-phosphorylated counterparts among plants. We retrieved the sequences of homologs of identified Arabidopsis phosphoproteins for two comparisons of different degree of divergence: (1) with five eudicot species (*Brassica pekinensis*, *Populustrichocarpa*, *Glycine max*, *Fragariaananassa* Duch and *Solanumlyco persicum*) and (2) with eight angiosperm species (five eudicots plus *Oryza sativa*, *Zea mays* and *Amborella trichopoda*). Then, the amino acid similarity was calculated according to the BLOSUM62 scoring system (Henikoff and Henikoff, 1992) in the homologs across eudicots and angiosperms. Both total identified phosphorylation sites and the phosphorylation sites on functionally important regulatory proteins were analysed to evaluate whether the functional phosphorylation sites were more evolutionarily conserved. The coefficient of conservation was represented by a decimal between 0 and 1. We set the residues with decimals more than 0.75 as highly conserved residues. The percentages of conserved phosphorylated residues were more than two times higher than that of random residues for both total phosphoproteins and regulatory proteins in both phases (Fig. 4C). This suggests that the phosphorylated residues are more conserved than their non-phosphorylated counterparts.

### Casein kinase II, MAPKs, and 14-3-3 proteins function as key regulators in anther development

Reversible protein phosphorylation is mediated by protein kinases and phosphatases, and different kinases preferentially phosphorylate specific substrates with conserved sequence motifs. To predict the relationship between the phosphorylation sites and the corresponding kinases during different anther phases, we performed the motif analysis using Motif-X to extract the over-represented patterns of amino acids from the input sequences containing phosphorylation sites (Schwartz and Gygi, 2005). In total we retrieved 45 over-represented patterns from the Ser/Thr-containing phosphopeptides with a significance value of *P*<0.000001 in our phosphoproteomic datasets (Supplementary Table S4 and Supplementary Fig. S5). Among them, 39 were phosphoSer motifs and six were phosphoThr motifs.

Plant casein kinase II (CKII), a ubiquitous Ser/Thr kinase localized in nuclear or cytoplasmic compartments, is involved in numerous biological processes during plant growth and development and mainly targets nuclear proteins, notably TFs (Moreno-Romero *et al.*, 2011). In our results, 1042 phosphorylation sites were the potential substrates of CKII, representing 26.7% of total identified phosphorylation sites. Different CKII isoforms have different roles during mammalian spermatogenesis: CKIIα functions in late spermatogenesis, and CKIIβ in early spermatogenesis (Mannowetz *et al.*, 2010). Moreover, CKII-defective Arabidopsis plants exhibit enhanced double-strand break repair rates and a reduced DNA damage response (Moreno-Romero *et al.*, 2012). This suggests that CKIIs and their substrates cooperate in a complex regulating network in anther development.

In Arabidopsis, MAPK3 and 6 potentially act downstream of the receptor-like kinases ER, ERL1 and ERL2 to regulate anther lobe formation (Hord *et al.*, 2008). Five hundred and forty-six phosphorylation sites detected here were in motifs that are promising substrates of MAPKs, providing further evidence at a post-translational level for the idea that MAPKs are crucial during anther development.

14-3-3 proteins are phosphoSer/Thr binding proteins that bind to targets with specific motifs that are involved in various biological processes such as stress responses, MAPK activation and apoptosis, and serving as adapters, activators and repressors (Denison *et al.*, 2011). In our results, 268 phosphorylation sites were possible binding targets of 14-3-3 proteins, suggesting that 14-3-3 proteins might serve as important regulators during anther development. Interestingly, the 14-3-3 binding motif R..sP is also the phosphorylation motif of the CDPK–SnRK superfamily (Baerenfaller *et al.*, 2011). It was found that a chimeric Ca^2+^/calmodulin-dependent protein kinase, a member of the CDPK–SnRK superfamily, was expressed at the meiotic stage during anther development in lily and tobacco (Liu *et al.*, 1998; Poovaiah *et al.*, 1999). Our results support a role of CDPK–SnRK kinases in coordinating phosphoprotein-binding 14-3-3 proteins to control anther development.

### Enrichment of transcriptional regulators among phosphoproteins in phase I

Transcription regulators (TRs) modulate transcription either as TFs or regulating the activities of TFs. There are 2286 Arabidopsis TFs in the Plant Transcription Factor Database (http://planttfdb.cbi.pku.edu.cn/index.php?sp=Ath). Anther development is controlled by a complex transcriptional regulatory network including numerous key TFs (Wijeratne *et al.*, 2007). Here we identified 474 TRs including 258 TFs. Among them, 107 TFs were identified as the non-phosphorylated form, whereas 151 were phosphorylated, including 104 phosphorylated only in anther phase I, 15 in phase II and 32 in both phases (Supplementary Table S5). In anther phase I, the percentages of both TRs and TFs in the phosphoproteomic dataset were more than two times higher than that in our proteomic dataset and the predicted Arabidopsis proteome. However, in phase II, the percentages of identified TRs and TFs among phosphoproteins were two times higher than that among the identified proteins from proteomic analysis, but only slightly higher than that in the Arabidopsis proteome (Fig. 2B).

**Fig. 5.**
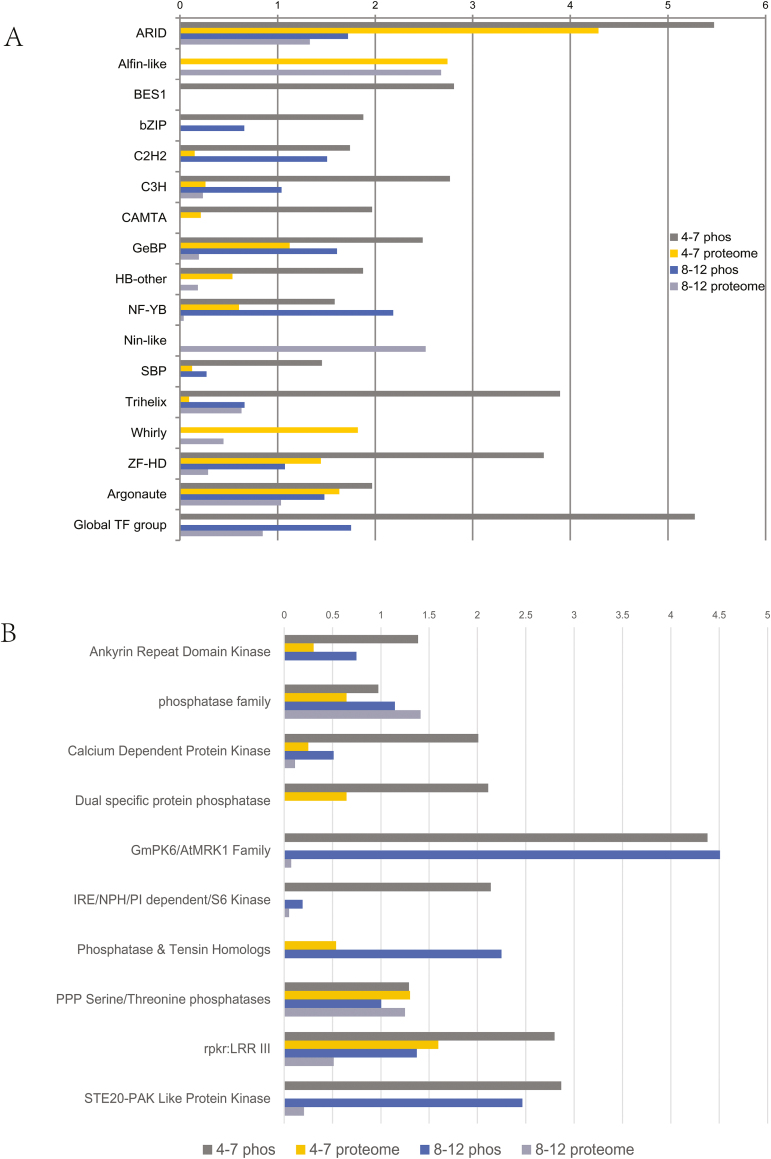
An overview of the TFs and kinases/phosphatases identified in proteome and phosphoproteome of developing anthers at phase I and II. (A) The over-represented TF families in the proteome and phosphoproteome of anther phases I and II, compared with the whole Arabidopsis genome (value >1.33 indicates *P*<0.05). The results showed that the phosphorylation patterns differ for TF families in different phases. (B) The enriched kinase or phosphatase families in proteome and phosphoproteome of anther phase I and II, compared with the whole Arabidopsis genome (value >1.33 indicates P<0.05).

To assess whether there is a difference in phosphorylation on TF families in different anther phases, we analysed TF families for enrichment using a one-side Fisher’s exact test of the hypergeometric distribution of the targeted sets (43 TF families from our results) compared with the background set (65 families in the Arabidopsis proteome) for identified proteins and phosphoproteins from the two phases. As shown in Fig. 5A, 17 TF families were over-represented (*P*<0.05) in our results. Members of three TF families were only detected in proteomic datasets, including Alfin-like, Nin-like and Whirly families; especially the Nin-like family was only detected in phase II. Three members in the BES1 family (BZR1, BZR2 and BEH4) were phosphorylated and detected in anther phase I but not phase II. Members of bZIP family were identified only as the phosphorylated form in both anther phases, suggesting that phosphorylation may play crucial roles in regulating the activities of bZIP TFs during anther development. The CAMTA family TFs were only observed in anther phase I, mainly in phosphorylated form, suggesting that phosphorylation may affect their activities.

### Protein kinases and phosphatases are preferentially phosphorylated in the anther

Protein kinases and phosphatases play important roles in plant development (Ganguly *et al.*, 2012). There are 1236 protein kinases and phosphatases according to the Arabidopsis Kinase Database (AtKD) (http://bioinformatics.cau.edu.cn/athKD/index.htm). Kinases are usually regulated by phosphorylation, either through autophosphorylation or by other kinases (Bogre *et al.*, 2003). Protein kinases and phosphatases were greatly over-represented in our phosphoproteomic datasets from both phases (6.29% and 4.53% for anther phases I and II, respectively) compared with the predicted proteome (3.49%) and proteins only from proteomic datasets for both phases (1.95% and 1.58%).

To estimate the phosphorylation extent of kinase families regulating the signal pathways during anther development, a kinase family enrichment analysis was performed on the identified protein datasets (59 kinase and phosphatase families from our results) compared with the background dataset (91 families in the Arabidopsis proteome) (Fig. 5B and Supplementary Table S6). Six kinase and four phosphatase families were enriched from our proteomic and phosphoproteomic datasets. The dual specific protein phosphatase family was observed only in the phosphoproteomic dataset of anther phase I, indicating the abundance of proteins in this family is low and phosphorylation might regulate their activities in anther phase I. Phosphatase and tensin homologs were detected in both anther phases but only in phosphorylated forms, suggesting that phosphorylation may affect their activities in both anther phases. Members of GmPK6/ArMRK1, IRE/NPH/PI dependent/S6 kinase and STE20-PAK like protein kinase families were also detected in both phases, while only the phosphorylated form was observed in phase I. Most members of the other five enriched families were detected in both proteomic and phosphoproteomic datasets from both phases.

### Phosphorylation might reduce activity of reactive oxygen species scavenging enzymes

Redox state, indicating the level of reactive oxygen species (ROS), was proposed as an important factor for specifying male germ cell fate in plants (Xing and Zachgo, 2008). In addition, the rice tapetum has a high ROS level just after meiosis, whereas the ROS level is reduced when the vacuolated microspore is formed (Hu *et al.*, 2011). It was thought that ROS promotes tapetal programmed cell death (PCD) during anther development (Zhang and Yang, 2014). In our results, one ROS producer and 41 ROS scavenging proteins were found in anther phase I and two ROS producing proteins and 47 ROS degrading proteins were found in phase II (Supplementary Table S7). Further analysis revealed that one ROS producer (respiratory burst oxidase homolog D; RBOHD) and four ROS scavenging enzymes (two thioredoxins (TRXs) and two glutaredoxins (GRXs)) were phosphorylated in the phase II, while none of the ROS-related proteins was phosphorylated in phase I.

NADPH oxidases, known to be ROS producers, belong to the respiratory burst oxidase homolog (RBOH) family and the Arabidopsis genome encodes ten RBOH family members. Phosphorylation and Ca^2+^ binding synergistically activate the ROS-producing enzyme activity of RBOHD in Arabidopsis (Ogasawara *et al.*, 2008). In our results, RBOHD was only phosphorylated in anther phase II; we therefore propose that the ROS-producing activity of RBOHD is only switched on in phase II. GRXs are small oxidoreductases that use glutathione to reversibly reduce disulfide bonds within proteins, affecting various cellular events and responses to oxidative stress (Lemaire, 2004). TRXs contain a conserved active site, WC(G/P)PC, and serve as a functional backup for GRXs. GRXs and TRXs regulate not only the switch from the mitotic cell cycle to meiosis but also later microspore formation (Dresselhaus and Franklin-Tong, 2013). Two Arabidopsis CC-type GRXs, ROXY1 and ROXY2, were shown to redundantly affect early anther development (Xing and Zachgo, 2008). A cystathionine β-synthase domain-containing protein CBSX2, which directly modulates TRXs in chloroplasts, regulates endothelial secondary cell wall thickening and anther dehiscence in Arabidopsis (Yoo *et al.*, 2011). In our results, ROS scavenging enzymes (41 in phase I and 47 in phase II) were found in both phases, while ROS scavenging enzymes (two TRXs and two GRXs) were only phosphorylated in phase II. During anther phase II, ROS is highly accumulated in tapetal cells and thus promotes tapetal PCD.

### Phosphorylation might regulate brassinosteroid signaling in early anther development

Brassinosteroids (BRs) regulate many processes of plant development, e.g. cell elongation, cell division, flowering time control, and responses to biotic and abiotic stresses (Wang, 2008). Recently, BRs were found to regulate male fertility through modulating the expression of key genes involved in Arabidopsis anther and pollen development (Ye *et al.*, 2010*b*) and BRs facilitate pollen development by directly promoting the expression of a rice MYB domain protein, CSA (Zhu *et al.*, 2015). As mentioned in the section on Mapman analysis, BR signaling components were significantly over-represented in the phosphoproteomic dataset of anther phases I, and we thus identified proteins relevant to BR signaling in our results (Supplementary Table S8). The negative regulators of the BR signaling pathway, a GSK3 family kinase BIN2 (Kim *et al.*, 2009), a transthyretin-like protein TTL (Nam and Li, 2004), three BZR TFs (Kim *et al.*, 2009) and another TF, HAT1 (Zhang *et al.*, 2014), were solely phosphorylated in anther phase I, suggesting that BR signaling might be regulated by phosphorylation in early anther development. However, the LRR-receptor kinase BSK1, the MAPKKK VIK (Ceserani *et al.*, 2009), four PP1-like phosphatase BSU1s, the 14-3-3 protein GF14 and the transcription elongation factor IWS1 (Li *et al.*, 2010) were phosphorylated in anther phase I. These proteins are positive regulators of the BR signaling pathway and their phosphorylation further supports a role for this form of post-translational modification in BR signaling.

## Discussion

### Four hundred and ninety-three novel phosphoproteins are largely anther specific

**Fig. 6.**
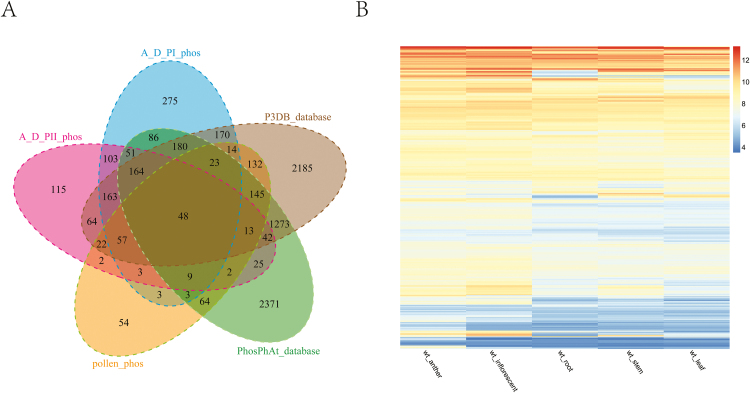
An overview of the 493 novel phosphoproteins from our developing anther phosphoproteome. (A) Overlap of the identified phosphoproteins in our developing anther phosphoproteome with phosphoproteins in the PhosPhAt database, the P^3^DB database and the mature pollen phosphoproteome (Mayank *et al.*, 2012). The 493 phosphoproteins were absent from pervious results. (B) Relative expression values across different tissues (data were retrieved from Ma *et al.*, 2012) (anther, inflorescent, stem, leaves and root) for 493 gene encoding phosphoproteins that are absent from the PhosPhAt database, the P^3^DB database and the mature pollen phosphoproteome. The 493 novel phosphoproteins are largely anther-enriched.

A comparative analysis was performed between our anther phosphoproteomic datasets and the PhosPhAt database (Heazlewood *et al.*, 2008), the P^3^DB database (Gao *et al.*, 2009) and the previously published pollen phosphoproteome (Mayank *et al.*, 2012) (Fig. 6A). A subset of 493 phosphoproteins were newly identified in our anther phosphoproteomic datasets (Supplementary Table S9). The previous datasets were generated from large-scale phosphoproteomic studies of tissues other than anthers, even not part of the inflorescence, suggesting that the 493 phosphoproteins might be largely specifically expressed or phosphorylated in the anther or other floral organs. To test this idea, we further evaluated the expression of genes for the 493 phosphoproteins in different Arabidopsis tissues (the gene expression data were retrieved from Ma *et al.*, 2012). As shown in Fig. 6B, the expression levels of most of the genes encoding the 493 phosphoproteins were higher in anther and inflorescence than in other tissues. Some genes also showed high expression levels in other tissues but the corresponding proteins have never been reported as phosphoproteins previously, suggesting that the phosphorylation of these proteins specifically occurred during anther development.

### Cross-talk of phosphorylation with transcriptional regulation and other post-translational modifications during anther development

Transcriptional regulation is a crucial regulatory mechanism controlling anther development, as genes encoding TFs have been demonstrated to be necessary for plant male fertility. In addition, transcriptomic analyses revealed that the mutation of TFs *spl*, *dyt1* or *ams* could obviously affect the expression levels of a subset of genes involved in receptor-like kinase signal transduction (Wijeratne *et al.*, 2007; Feng *et al.*, 2012; Ma *et al.*, 2012), and meanwhile the expression levels of a number of TF genes were also up/down-regulated in plants mutated for the *EMS1* gene encoding a receptor-like kinase (Wijeratne *et al.*, 2007). Therefore, TFs could influence post-translational modifications through up/down-regulating the expression of receptor-like kinase genes, and conversely receptor-like kinases might affect the activities of TFs through post-translational modification. In our results, 149 TFs were phosphorylated, which supported that protein phosphorylation did regulate the transcriptional regulation through affecting the activities of TFs during anther development at the post-translational regulation level.

In our phosphoproteomic analysis, we found that numerous proteins involved in other post-translational modifications were phosphorylated. For instance, there were 15 phosphoproteins involved in acetylation/deacetylation, ten in DNA/protein methylation, four in protein sumoylation and 32 in ubiquitination (Supplementary Table S10). This provides evidence that protein phosphorylation regulates other processes from the transcriptional to post-translational levels. Further comparative analysis of transcriptomic datasets on mutants of *ASK1*, encoding a ubiquitin-dependent protein kinase, and *SDS*, encoding a meiotic cyclin-like protein (Ma *et al.*, 2012), proteomic dataset of *ask1* mutant (in press) and our phosphoproteomic results showed that protein products of numerous up/down-regulated genes were phosphorylated. This suggests phosphorylation may regulate or coordinate other post-translational modifications during anther development.

### Phosphorylation of products of genes required for anther development

In this study, 26 proteins known to be involved in Arabidopsis anther development were identified from proteomic and phosphoproteomic analyses of two anther phases. Among them, six proteins were phosphorylated at a total of 11 sites (Supplementary Tables S2 and S11). Interestingly, the LRR-receptor-like kinase BAM1 was phosphorylated in both phases (at S996 in phase I and S516, S989, S992 and S996 in phase II), whereas phosphorylated BAM2 (at S517) was only detected in phase II. It has been reported that BAM1 and BAM2 together regulate the specification of somatic cells during early anther development (Hord *et al.*, 2006). We therefore proposed that BAM1 and BAM2 might also have important functions during late anther development, and BAM2 may only function as a substitute of BAM1 (when *BAM1* is mutated) in early anther development. In a similar situation, MAPK6 was also phosphorylated in both phases (at T221 and Y223), whereas ER was only phosphorylated in phase I (at S975), indicating that MAPK6 may play important roles throughout anther development while ER primarily functions only during early anther development (Hord *et al.*, 2008). EMS1 was reported to function as the phosphorylated form during early anther development even though the phosphorylation site remains unknown (Canales *et al.*, 2002; Zhao *et al.*, 2002), but EMS1 was only observed as the unphosphorylated form in our experiment. This might be due to the fact that our materials only included anther stages 4–12, EMS1 may function before stage 4, or the phosphorylation level is too low to be detected. TGA9 (phosphorylated at S41 and S42), which is involved in regulating stamen morphogenesis (Murmu *et al.*, 2010), and an ABC transporter, WBC27 (phosphorylated at S517), controlling pollen wall formation and patterning (Dou *et al.*, 2011), were phosphorylated in phase II, suggesting that phosphorylation might regulate the activities of TGA9 and WBC27 in late anther development.

Meiosis is essential for the formation of functional gametes and previous studies found that protein phosphorylation plays crucial roles in animal meiosis (Woglar *et al.*, 2013; Murakami and Keeney, 2014). In our results, 21 known meiotic proteins were identified, and among them, nine were phosphorylated at 37 sites (Table 1 and Supplementary Table S11). Phosphorylation of SCC3 and RBR1 were detected in both phases. Six known meiotic proteins were solely phosphorylated in phase I, and these are important for chromosome condensation (CHR4), DNA double-strand break repair (MEI1, ATLIG4 and PDS5), crossover formation (RFC1) and chromosome segregation (WAPL1). A member of the mei2-like gene family, AML5, is involved in formation of SPO11-1-independent chromosome fragmentation and was phosphorylated in phase II, after meiosis is completed. This suggests that either phosphorylation negatively regulates its activity or it may have functions other than in meiosis during late anther development.

**Table 1.** Anther development and meiosis related phosphoproteins identified in phosphoproteome of anther phases I and II

**AGI**	**Gene symbol**	**Function**	**Reference**	**4–7 phos**	**8–12 phos**	**Phospho-site in stages 4–7**	**Phospho-site in stages 8–12**
**Anther development**							
AT2G26330	ER	Anther lobe development	Hord *et al.* (2008)	+	–	S975	
AT2G43790	MPK6	Anther lobe development	Hord *et al.* (2008)	+	+	T221, Y223	T221, Y223
AT5G65700	BAM1	Somatic cell differentionation	Hord *et al.* (2006)	+	+	S996	S516, S989, S992, S996
AT1G08320	TGA9	Anther lobe development	Murmu *et al.* (2010)	–	+		S123
AT3G13220	WBC27	Transport of sporopollenin precursors	Dou *et al.* (2011)	–	+		S41, S42
AT3G49670	BAM2	Somatic cell differentiation	Hord *et al.* (2006)	–	+		S517
**Meiosis**							
AT1G11060	WAPL1	Meiotic chromosome segregation	De *et al.* (2014)	+	–	S929	
AT1G77320	MEI1/MCD1	Independent of SPO11 DSB recombination repair	Grelon *et al.* (2003)	+	–	S884	
AT2G47980	SCC3	Meiotic cohesion and chromosome segregation	Chelysheva *et al.* (2005)	+	+	S22, S31, S40, T814	S31, S1053, S1055, S1060, S1062, S1065
AT3G12280	RBR1	Synapsis, reduction of COs	Kurzbauer and Schlogelhofer (2011)	+	+	S885, S898	S885
AT5G22010	RFC1	Crossover formation	Wang *et al.* (2012)	+	–	S882	
AT5G44800	CHR4	Chromosome condensation	Lee *et al.* (2010)	+	–	S331, S1354, T1422, S1423, T1975, S2138	
AT5G47690	PDS5	Chromatin structure, chromosome segregation	Losada *et al.* (2005)	+	–	S1131, S1141, S1250, S1274, S1281, S1284, S1289, S1299, S1518, S1548, S1549, S1562, S1584	
AT5G57160	ATLIG4	Double-strand break repair via nonhomologous end joining	Heacock *et al.* (2007)	+	–	S994, S997, S1058	
AT1G29400	AML5	Chromosome fragmentation	Kaur *et al.* (2006)	–	+		S792

+, the protein was identified; –, the protein was not identified; 4–7 phos, the phosphoproteome of developing anthers at stages 4–7 (phase I); 8–12 phos, the phosphoproteome of developing anthers at stages 8–12 (phase II).

### Phosphorylation regulatory network during anther development

Phosphorylation regulation is a dynamic process, and different kinases may function in different anther stages. To assess whether there is a preference of active kinase families during anther development, we performed a comparative analysis between the active (phosphorylated) kinase families identified in two phases and mature pollen (Mayank *et al.*, 2012) (Supplementary Table S12). Interestingly, LRR-RLK family members were only phosphorylated in the anther, consistent with the previous findings that several members of the LRR-RLK family, e.g. EMS1, BAM1/2, SERK1/2 and ER/ERL1/ERL2, play critical roles in early anther development. CRPK-RLK and RLCK VII subfamilies were phosphorylated in mature pollen, which is supported by genetic evidence that CRPK-RLK family members FERRINIA, THESEUS1 and ANX1/2 are preferentially expressed in pollen and regulate pollen tube growth (Boisson-Dernier *et al.*, 2011; Kessler and Grossniklaus, 2011).

**Fig. 7.**
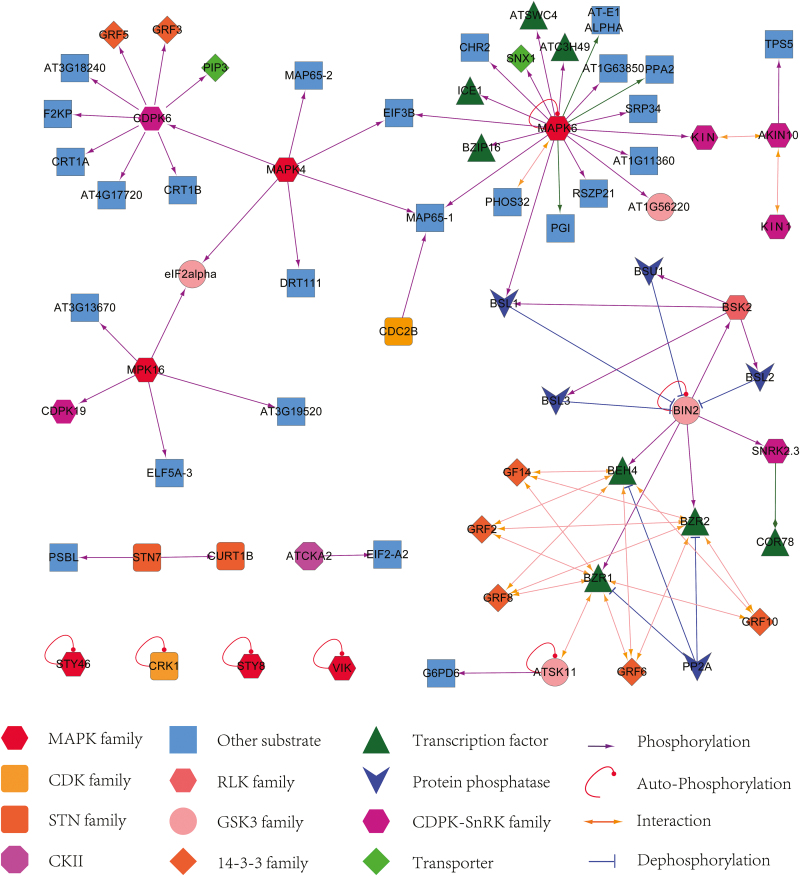
The kinase–substrate network extracted from the phosphoproteome of anther phase I. The results are color-coded to indicate the kinase and its substrate where the putative substrates of MAPK6 were found to be over-represented. Triangular arrowhead represents phosphorylation regulation, T-arrowhead indicates dephosphorylation regulation, and circular arrowhead indicates autophosphorylation of kinase.

To investigate the possible regulatory network mediated by protein phosphorylation, we predicted the kinase–substrate relationships according to the results from motif analysis and the PhosPhAt database (Zulawski *et al.*, 2013). The analysis supported 84 and 33 predicted kinase–substrate pairs in the phosphoproteome of phase I and II, respectively, allowing the proposed interaction network illustrated with Cytoscape 2.8 (Fig. 7, Supplementary Fig. S6 and Supplementary Table S13).

The putative substrates of MAPK6 were most highly enriched in both phases, where 20 and 18 substrates were identified in phase I and II, respectively (Fig. 7, Supplementary Fig. S6). This is consistent with essential roles of MAPK6 in anther development (Hord *et al.*, 2008). Among the putative substrates of MAPK6, three TFs (bZIP16, the myb-like TF ATSWC4, and the C3H family TF ATC3H49) were identified in both phases, suggesting an important role of TF phosphorylation throughout anther development. ICE1, a bHLH family TF, was only phosphorylated at S403 in phase I. A previous study uncovered that phosphorylation of ICE1 by the SnRK2 family kinase OST1 at S278 stabilized the ICE1 protein during cold tolerance (Ding *et al.*, 2015). This suggests that phosphorylation of ICE1 by MAPK6 may affect its activity in phase I. Substrates of CDPK6, MAPK4 and MAPK16 were also over-represented in our network of predicted kinase-to-target relationships, suggesting these kinases may also play important roles in phase I. COR78 was assigned as the target of SnRK2.3 in both phases. SnRK2.3 is a member of the SNF1-related kinases regulating numerous metabolic and transcriptional pathways in response to energy deprivation and ABA signals (Polge and Thomas 2007; Baena-Gonzalez and Sheen 2008). It contains a catalytic domain similar to that of SNF1 in yeast and AMPK in animals (Emanuelle *et al.*, 2015). It was found that the barley SnRK1α and SnRK1β are expressed in anthers and the *snrk1* mutant caused abnormal pollen development and male sterility (Zhang *et al.*, 2001). We therefore propose that SnRK2 might also be involved in anther development. The BR signal pathway regulatory network was also enriched in the kinase–substrate network of phase I; however, most of the negative regulators of BR signals were phosphorylated, implicating that BR signals may be hampered in early anther development.

## Conclusion

In flowering plants, anther development is a crucial process that directly determines the male fertility of the plant. Great efforts have been made in unraveling the signaling and regulatory mechanisms of anther development by genetic or transcriptomic approaches. Numerous issues remain unclear since anther development is a dynamic and complicated process. Therefore, the data obtained here from anther phases I and II provide an insight into the post-translational modification in different phases and enrich the resources for investigating the role of reversible phosphorylation in anther development. Some of the key anther development proteins were phosphorylated, which provides a clue for the possible molecular mechanisms for their regulation of male fertility. Hundreds of novel phosphoproteins found in our study are strongly enriched in the anther based on the genome-wide expressing profiles, which might be further explored for their potential roles in anther development.

## Supplementary data

Supplementary data are available at *JXB* online.

Figure S1. Mapman analysis of total identified proteins (including phosphoproteins). Only functional bins with *P<*1.0×10^–2^ are presented.

Figure S2. Overlap of proteomic and phosphoproteomic analyses in anther phase I and II.

Figure S3. The enriched MapMan bins (bin numbers and bin names) of identified phosphoproteins (proteins identified in proteomic analysis were set as background) for anther phase I and II. Only functional bins with *P<*1.0×10^–2^ for identified proteins are presented.

Figure S4. The enriched MapMan bins (bin numbers and bin names) of proteomic datasets for anther phase I and II. Only functional bins with *P<*1.0×10^–2^ for identified proteins are presented.

Figure S5. Phosphorylation motifs of phosphoSer and phosphoThr predicted by Motif-X.

Figure S6. The kinase–substrate network extracted from phosphoproteome of anther phase II.

Table S1. Identified total peptides and phosphopeptides in proteomic and phosphoproteomic analyses of anther phase I and II.

Table S2. Identified proteins (including phosphoproteins) in anther phase I and II.

Table S3. Overview of phosphoproteins identified in anther developing phase I and II.

Table S4. Phosphorylation motifs enriched by motif-X and putative protein kinase or binding proteins.

Table S5. Overview of identified transcription regulators in proteomic and phosphoproteomic analyses of anther phase I and II.

Table S6. Overview of identified receptor like kinases in proteomic and phosphoproteomic analyses of developing anther and pollen.

Table S7. Identified ROS related proteins in proteome and phosphoproteome analyses of developing anthers.

Table S8. Identified BR signal proteins in anther phase I and II.

Table S9. Overview of 493 novel phosphoproteins.

Table S10. Overview of identified anther development and meiosis key proteins.

Table S11. Comparison of phosphorylated kinase between developing anthers and mature pollens.

Table S12. Identified phosphoproteins involved in other protein/DNA modifications

Table S13. List of predicated kinase to substrate relationships in phosphoproteomic analyses of two anther phases.

Supplementary Data
