# Supplementary material for: Abundant protein phosphorylation potentially regulates Arabidopsis anther development
Source: J Exp Bot. 2016 Aug 16;67(17):4993–5008. doi: 10.1093/jxb/erw293 (PMC5014169; doi:10.1093/jxb/erw293)
Supplement: Supplementary Data [file supp_67_17_4993__index.html]

Abundant protein phosphorylation potentially regulates Arabidopsis anther development — Abundant protein phosphorylation potentially regulates Arabidopsis anther development — Supplementary Data 

# Abundant protein phosphorylation potentially regulates Arabidopsis anther development

## Supplementary Data

Data files

- supplementary\_figures\_S1\_S6.pdf - Supplementary Data
- supplementary\_table\_S1.xlsx - Supplementary Data
- supplementary\_table\_S2.xlsx - Supplementary Data
- supplementary\_table\_S3.xlsx - Supplementary Data
- supplementary\_table\_S4.xlsx - Supplementary Data
- supplementary\_table\_S5.xlsx - Supplementary Data
- supplementary\_table\_S6.xlsx - Supplementary Data
- supplementary\_table\_S7.xlsx - Supplementary Data
- supplementary\_table\_S8.xlsx - Supplementary Data
- supplementary\_table\_S9.xlsx - Supplementary Data
- supplementary\_table\_S10.xlsx - Supplementary Data
- supplementary\_table\_S11.xlsx - Supplementary Data
- supplementary\_table\_S12.xlsx - Supplementary Data
- supplementary\_table\_S13.xlsx - Supplementary Data
