# Supplementary material for: Abundant protein phosphorylation potentially regulates Arabidopsis anther development
Source: J Exp Bot. 2016 Aug 16;67(17):4993–5008. doi: 10.1093/jxb/erw293 (PMC5014169; doi:10.1093/jxb/erw293)
Supplement: Supplementary Data [file supp_erw293_supplementary_figures_S1_S6.pdf]

**Abundant Protein Phosphorylation Potentially Regulates Arabidopsis Anther Development**

Juanying Ye, Zaibao Zhang, Chenjiang You, Xumin Zhang, Jianan Lu, Hong Ma*

[1]State Key Laboratory of Genetic Engineering and Collaborative Innovation Center of Genetics and Development, Institute of Plant Biology, School of Life Sciences, Fudan University, Shanghai 200433, China

**Supplemental Figure legends.**

**Figure S1.** Mapman analysis of total identified proteins (including phosphoproteins). Only functional bins with $P<1.0\times10^{-2}$ are presented.

**Figure S2.** Overlap of proteomic and phosphoproteomic analyses in anther phase I and II. (a) Overlap between phosphoproteome (4-7phos) and proteome (4-7proteome) at anther stage 4-7 (phase I); (b) Overlap between phosphoproteome (8-12phos) and proteome (8-12proteome) at anther stage 8-12 (phase II).

**Figure S3.** The enriched MapMan bins (bin numbers and bin names) of identified phosphoproteins (proteins identified in proteomic analysis were set as background) for anther phase I (a) and II (b). Only functional bins with $P<1.0\times10^{-2}$ for identified proteins are presented.

**Figure S4.** The enriched MapMan bins (bin numbers and bin names) of proteomic datasets for anther phase I and II. Only functional bins with $P<1.0\times10^{-2}$ for identified proteins are presented.

**Figure S5.** Phosphorylation motifs of phosphoSer and phosphoThr predicted by Motif-X.

**Figure S6.** The kinase-substrate network extracted from phosphoproteome of anther phase II.

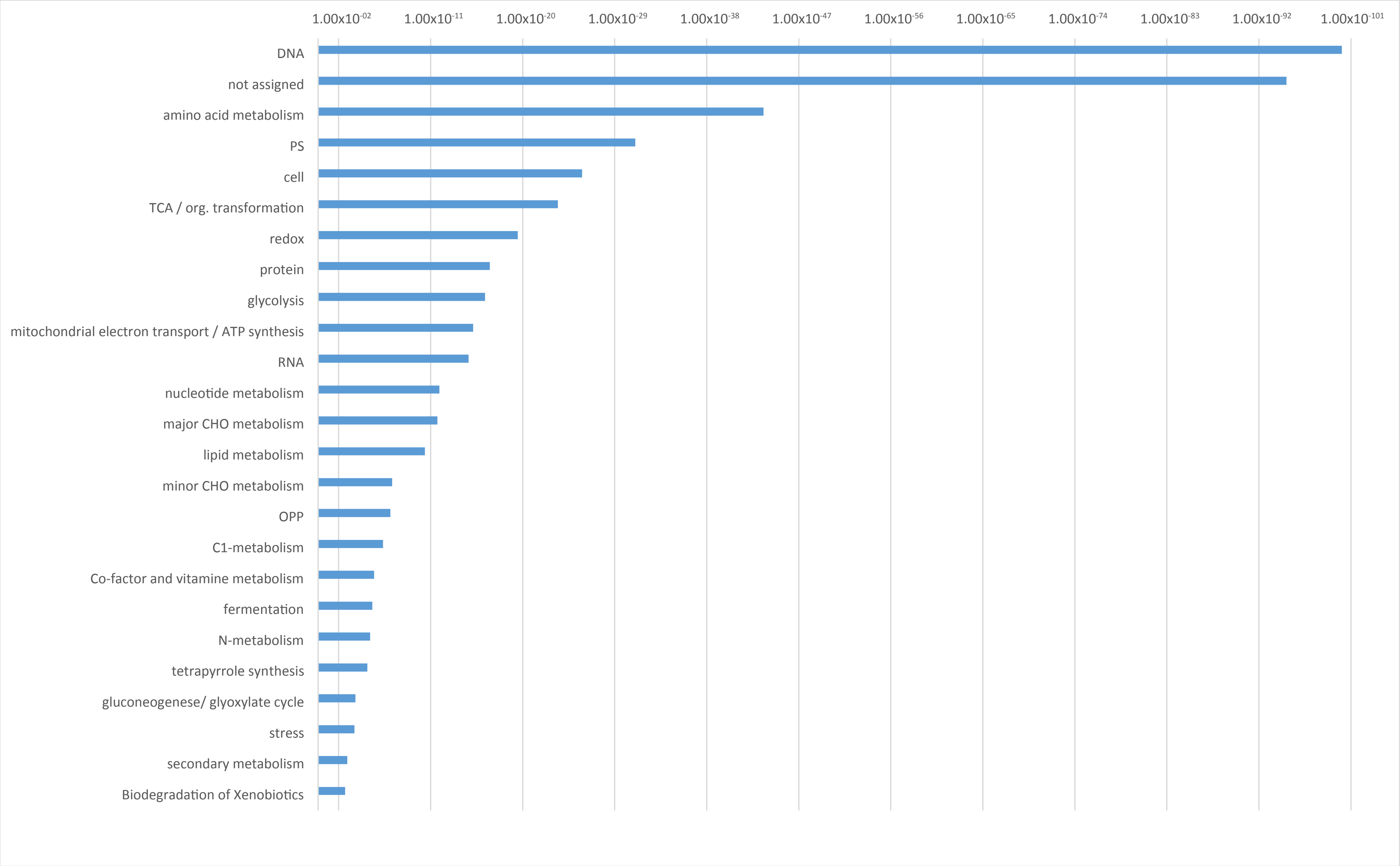
1.00x10-02
1.00x10-11
1.00x10-20
1.00x10-29
1.00x10-38
1.00x10-47
1.00x10-56
1.00x10-65
1.00x10-74
1.00x10-83
1.00x10-92
1.00x10-101
DNA
not assigned
amino acid metabolism
PS
cell
TCA / org. transformation
redox
protein
glycolysis
mitochondrial electron transport / ATP synthesis
RNA
nucleotide metabolism
major CHO metabolism
lipid metabolism
minor CHO metabolism
OPP
C1-metabolism
Co-factor and vitamine metabolism
fermentation
N-metabolism
tetrapyrrole synthesis
gluconeogenese/ glyoxylate cycle
stress
secondary metabolism
Biodegradation of Xenobiotics

a

b

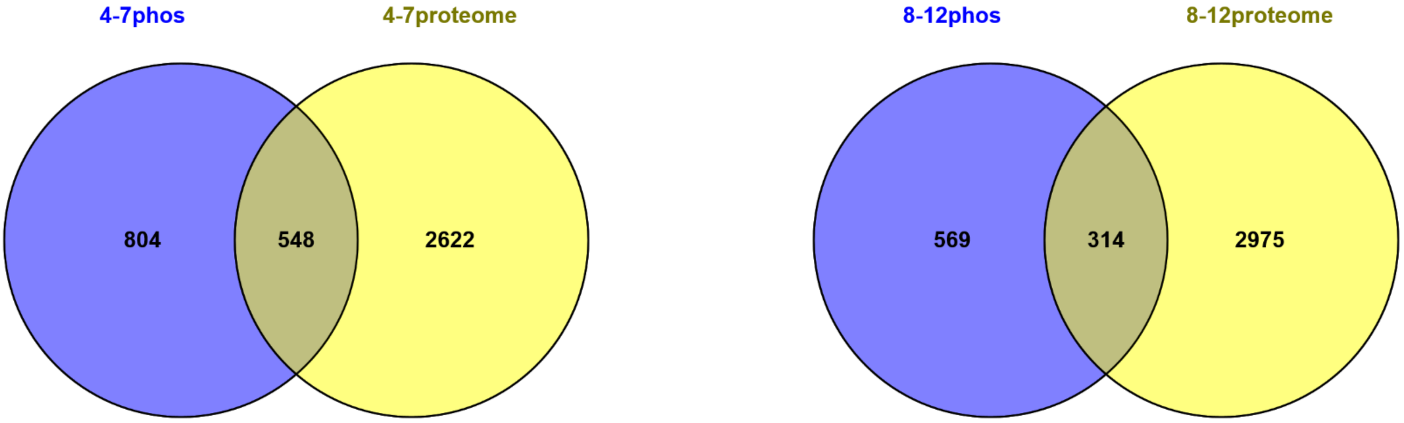
4-7phos
4-7proteome
804
548
2622
8-12phos
8-12proteome
569
314
2975

a

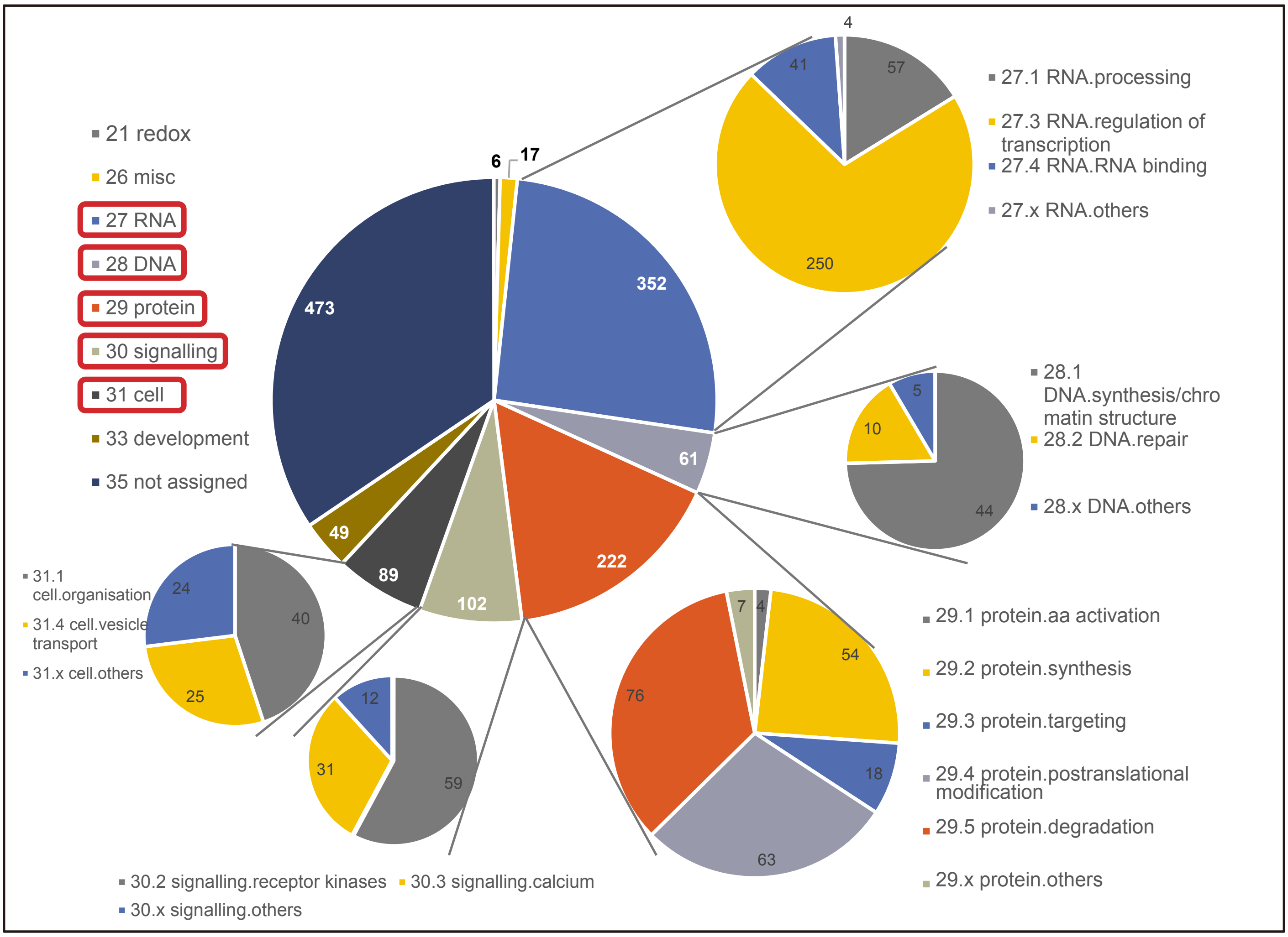

21 redox
26 misc
27 RNA
28 DNA
29 protein
30 signalling
31 cell
33 development
35 not assigned
473
352
222
102
89
61
49
6
17
27.1 RNA.processing
27.3 RNA.regulation of transcription
27.4 RNA.RNA binding
27.x RNA.others
4
57
41
250
28.1 DNA.synthesis/chromatin structure
28.2 DNA.repair
28.x DNA.others
5
10
44
29.1 protein.aa activation
29.2 protein.synthesis
29.3 protein.targeting
29.4 protein.postranslational modification
29.5 protein.degradation
29.x protein.others
7
4
54
76
18
63
31.1 cell.organisation
31.4 cell.vesicle transport
31.x cell.others
24
40
25
12
31
59
30.2 signalling.receptor kinases
30.3 signalling.calcium
30.x signalling.others


b

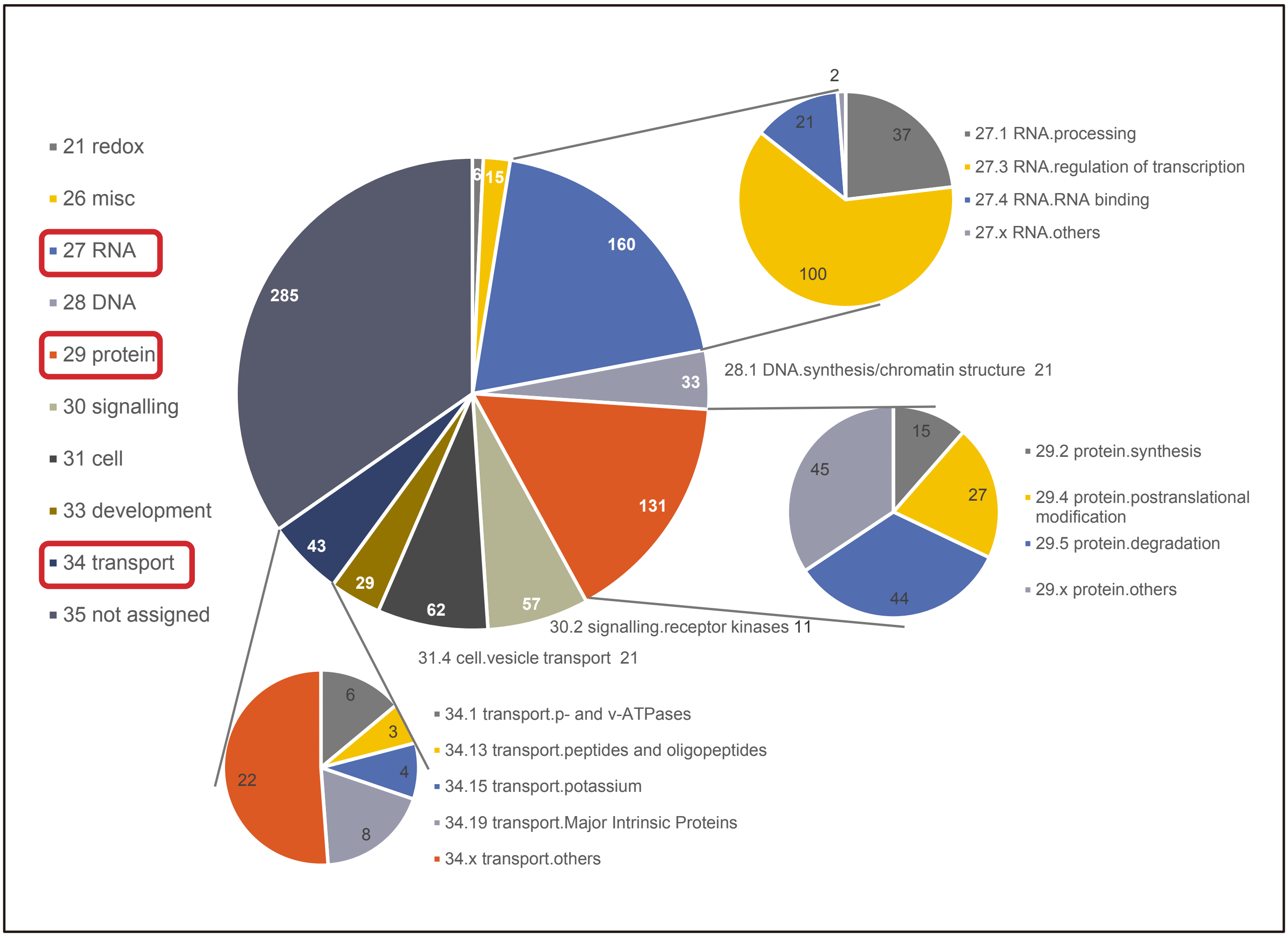

21 redox
26 misc
27 RNA
28 DNA
29 protein
30 signalling
31 cell
33 development
34 transport
35 not assigned
285
160
131
62
57
43
33
29
6
15
2
37
21
100
27.1 RNA.processing
27.3 RNA.regulation of transcription
27.4 RNA.RNA binding
27.x RNA.others
28.1 DNA.synthesis/chromatin structure 21
15
27
44
45
29.2 protein.synthesis
29.4 protein.postranslational modification
29.5 protein.degradation
29.x protein.others
30.2 signalling.receptor kinases 11
31.4 cell.vesicle transport 21
6
3
4
8
22
34.1 transport.p- and v-ATPases
34.13 transport.peptides and oligopeptides
34.15 transport.potassium
34.19 transport.Major Intrinsic Proteins
34.x transport.others

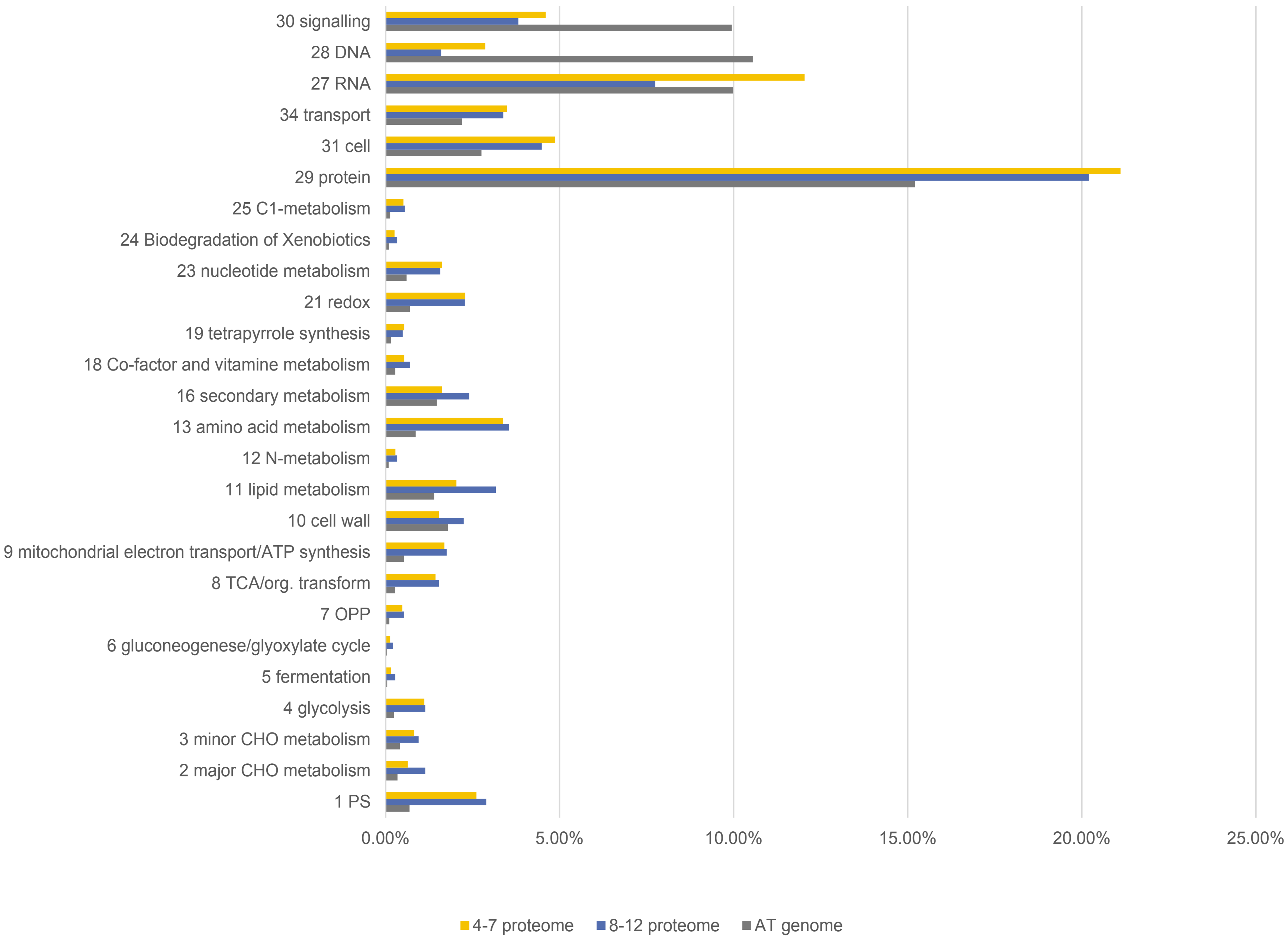

30 signalling
28 DNA
27 RNA
34 transport
31 cell
29 protein
25 C1-metabolism
24 Biodegradation of Xenobiotics
23 nucleotide metabolism
21 redox
19 tetrapyrrole synthesis
18 Co-factor and vitamine metabolism
16 secondary metabolism
13 amino acid metabolism
12 N-metabolism
11 lipid metabolism
10 cell wall
9 mitochondrial electron transport/ATP synthesis
8 TCA/org. transform
7 OPP
6 gluconeogenese/glyoxylate cycle
5 fermentation
4 glycolysis
3 minor CHO metabolism
2 major CHO metabolism
1 PS
0.00%
5.00%
10.00%
15.00%
20.00%
25.00%
4-7 proteome
8-12 proteome
AT genome

Motif 1-15

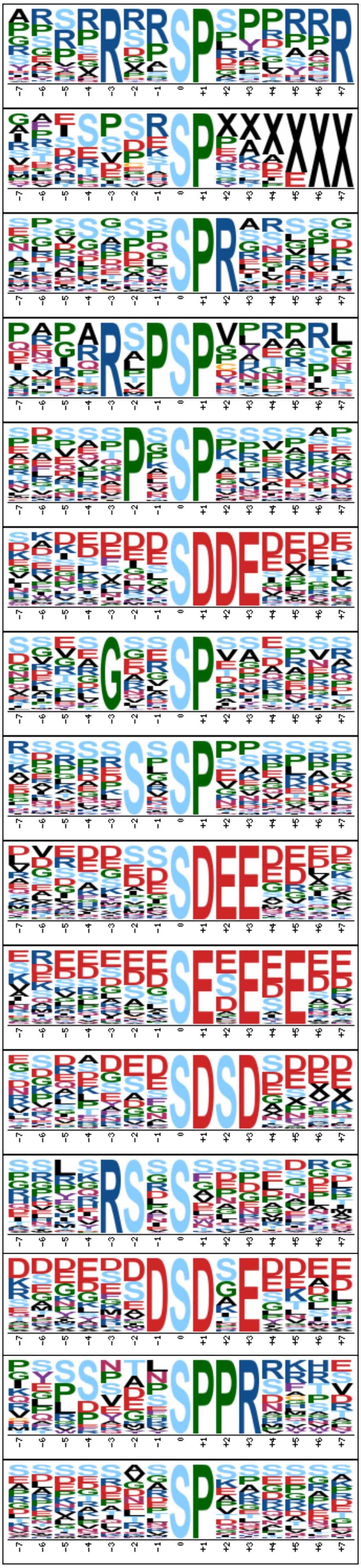

Motif 15-30

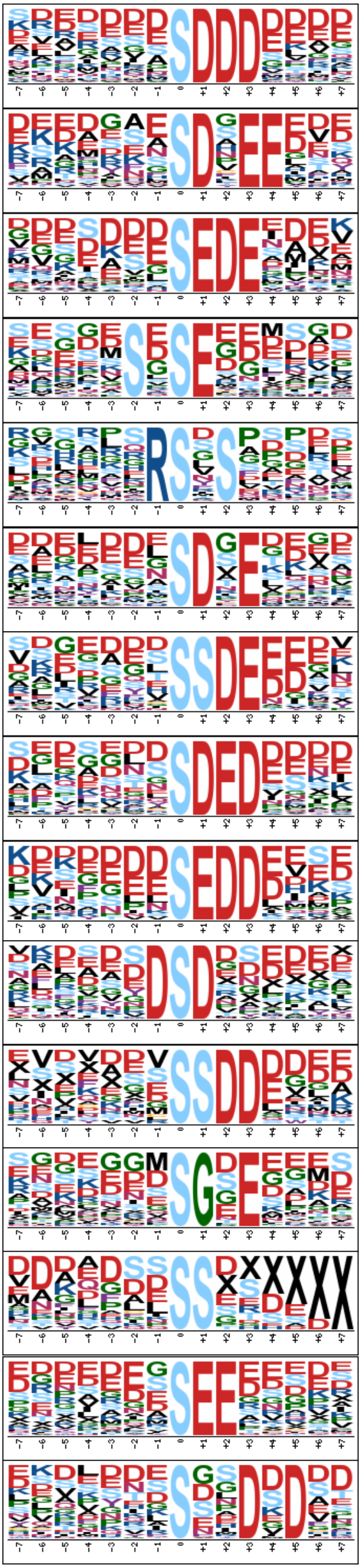

Motif 30-45

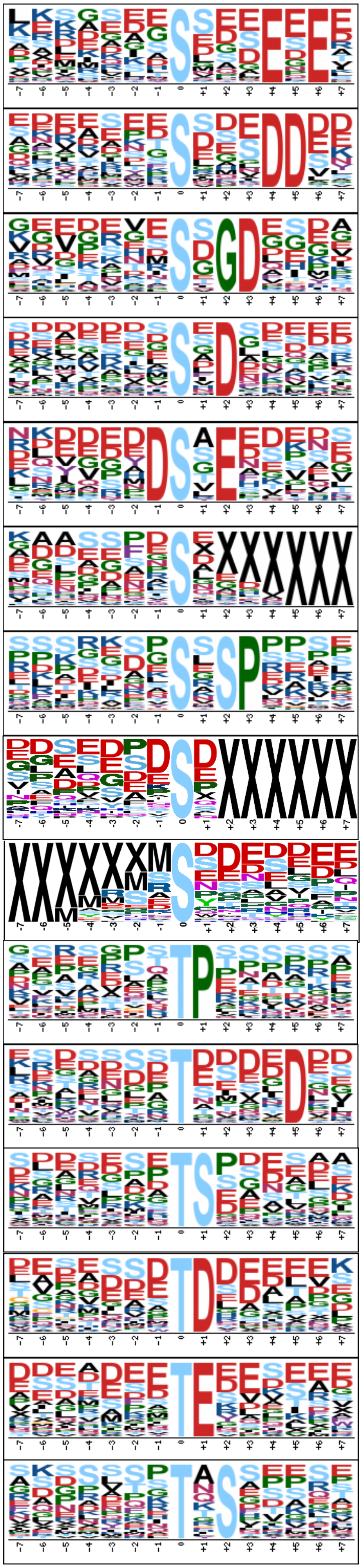

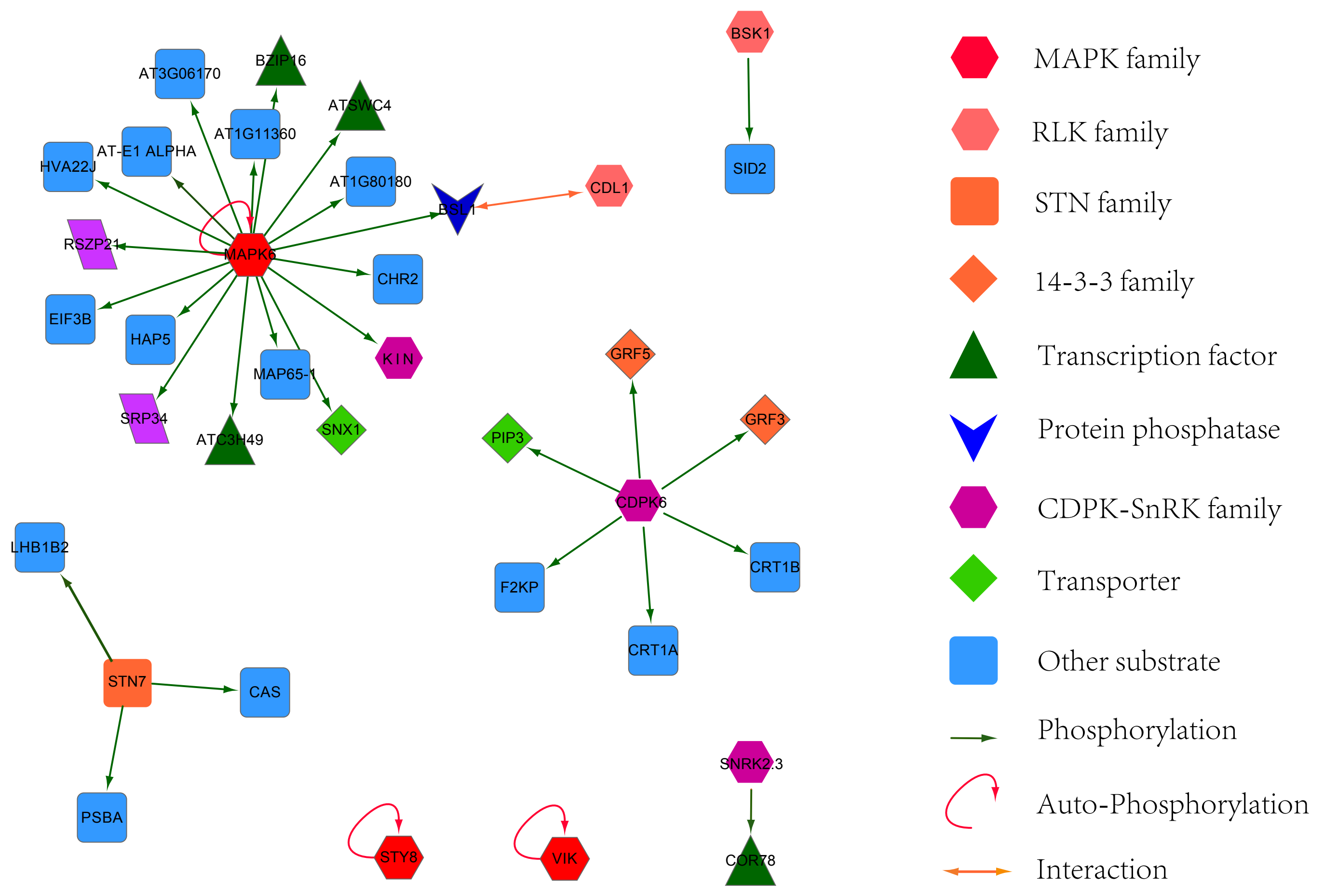

BSK1
SID2
AT3G06170
BZIP16
ATSWC4
AT1G11360
AT-E1 ALPHA
HVA22J
AT1G80180
BSL1
CDL1
RSZP21
MAPK6
CHR2
EIF3B
HAP5
KIN
MAP65-1
SRP34
ATC3H49
SNX1
GRF5
GRF3
PIP3
CDPK6
LHB1B2
F2KP
CRT1B
CRT1A
STN7
CAS
PSBA
SNRK2.3
STY8
VIK
COR78
MAPK family
RLK family
STN family
14-3-3 family
Transcription factor
Protein phosphatase
CDPK-SnRK family
Transporter
Other substrate
Phosphorylation
Auto-Phosphorylation
Interaction
